# Developing Policy for the Healthy Life Trajectories Initiative: Going from National to International

**DOI:** 10.1089/bio.2022.0198

**Published:** 2023-06-19

**Authors:** Dimitri Patrinos, Erika Kleiderman, William Fraser, Ma'n H. Zawati

**Affiliations:** ^1^ Centre of Genomics and Policy, Department of Human Genetics, Faculty of Medicine and Health Sciences, McGill University, Montreal, Quebec, Canada.; ^2^ Centre de recherche du Centre Hospitalier Universitaire de Sherbrooke, Sherbrooke, Quebec, Canada.; ^3^ Department of Obstetrics and Gynecology, Université de Sherbrooke, Sherbrooke, Quebec, Canada.

**Keywords:** governance, data access, biosample access, noncommunicable diseases, policy

## Abstract

**Background:**

Scientific research is becoming an increasingly collaborative and global venture. The Healthy Life Trajectories Initiative (HeLTI), for instance, is an international Developmental Origins of Health and Disease research collaboration developed to address the increasing burden of noncommunicable diseases around the world. It comprises four separate but harmonized cohort trials in Canada, China, India, and South Africa. These cohorts will generate rich data and biosample sets that can be shared both within the HeLTI Consortium and with other researchers from around the world.

**Methods:**

To ensure the coordination and operation of these types of collaborative research initiatives, a standardized and harmonized governance model is required to regulate the processes and interactions between all involved actors. To develop the governance models, frameworks and related policies from other longitudinal cohort studies and biobanks were used, as were guidance documents on biobank and database governance and relevant literature on data and biobank governance.

**Results:**

This article outlines the key components of the governance model for the HeLTI Consortium, including management of the cohorts' respective databases and biobanks, access to data and biosamples, and considerations related to intellectual property and publications.

**Conclusion:**

Governance within international collaborative research ventures is critical to ensure the operations and benefits of these types of research apparatuses. Although this article focuses on the HeLTI Consortium as a model, it may nonetheless serve as a model for both current and future collaborative consortium-based research initiatives.

**Clinical Trial Registration Numbers:**

Canada, ISRCTN13308752; China, ChiCTR1800017773; India, ISRCTN20161479; South Africa, PACTR201903750173871.

## Introduction

The Healthy Life Trajectories Initiative (HeLTI) is an international Developmental Origins of Health and Disease research collaboration developed to address the increasing burden of noncommunicable diseases (NCDs) around the world, including obesity, diabetes, cardiovascular disease, and mental health conditions. HeLTI consists of four separate but harmonized cohort trials in South Africa (Soweto),^1^ India (Mysore),^2^ China (Shanghai),^3^ and Canada (Alberta and Ontario).^4^

All cohorts, collectively known as the HeLTI Consortium, are focused on developing evidence-based interventions that span four developmental periods: (1) preconception, (2) pregnancy, (3) infancy (0–2 years), and (4) early childhood (3–5 years). The overall goal of the HeLTI Consortium is to reduce risk factors for NCDs—focusing mainly on childhood obesity, through health promotion in the preconception, prenatal, and childhood periods—and ultimately, to benefit the life-long health and well-being of the child.

In furtherance of this goal, cohorts will collect an array of data and biosamples from both mothers and children. Data sets will include basic health measures (e.g., blood pressure, height, weight, and body composition), health history, and lifestyle choices. Biosamples collected will include blood, urine, stool, and saliva from mothers, as well as umbilical cord blood and placenta from children. Each cohort will recruit between 4000 and 8000 participants, with data and biosample collection expected to continue through 2025.^5^

Rich data and biosamples will, therefore, be collected to promote research on the social and biological pathways of health and disease. Ultimately, this research will help inform policy and decision making on the improvement of health and the prevention and treatment of NCDs. To help translate research into policy action, a prospective governance model needed to be developed that addressed the key principles and elements of the HeLTI Consortium.

Governance can be defined as the “process of policy making and management that guides and oversees research in a consistent and structured manner.”^6^ Governance frameworks regulate the interactions between the various stakeholders involved in a research project and contain all the agreements, policies, and procedures that are key to decision making and ensuring accountability.^7^ Good governance is essential for maintaining public trust in research, ensuring the protection of research participants' rights, and maintaining the availability of data and biosamples for research.^7^ The lack of a governance framework can impede the research-conducting capacity of the research enterprise, most notably in the context of international collaborative initiatives such as HeLTI.^7^

Indeed, scientific research is becoming increasingly global in nature, and data and biosamples can now be shared within a collaborative network of research teams located in different countries.^8^ Research consortia now bring together investigators from around the world, studying areas such as rare diseases,^9^ child psychopathology,^10^ and cardiovascular health.^11^ NCD-related studies, however, have largely targeted specific regions or countries.^12^ HeLTI differs from these previous initiatives by linking cohorts from four different countries. Although this creates new opportunities through increased benefit sharing, especially between developed and developing countries,^13,14^ it can also lead to “legal fragmentation,” whereby a complex and heterogeneous patchwork of laws must be navigated.^13,15^

With this increased focus on international collaboration, the importance of standardized and harmonized policies and procedures across countries is critical. Uniform governance mechanisms applicable across all levels within an international research consortium are key to its optimal utility and operation. Accordingly, this article describes our approach to developing the governance frameworks for the HeLTI Consortium. This included an overarching international framework for the consortium, as well as individual frameworks for each cohort.

This article highlights the key elements in the development of these frameworks and important lessons learned throughout. Our discussion will, therefore, be limited to these key elements and will not address other important issues addressed in the governance frameworks, such as informed consent, return of research results, and incidental findings.

Our article begins with context concerning the governance frameworks and the steps that were taken in their development. Next, we describe the key principles and elements of the governance frameworks: database and biobank management, data and biosample sharing, publications, and intellectual property. Finally, we conclude with the key lessons we learned during the development of the governance frameworks, which may serve as models for similar initiatives. As research becomes increasingly international and collaborative, the HeLTI model can serve as an example for the development of future governance frameworks.

## Methods: Context for the HeLTI International and Cohort-Specific Governance Frameworks

Cohorts had limited experience in data and biosample governance. A group of ethics and policy experts based at the Centre of Genomics and Policy (CGP) of McGill University was, therefore, mandated to review and provide ethical and legal guidance on the cohorts' informed consent forms (ICFs) and study protocols before their submission for review by their institutional review boards.

In addition to reviewing this documentation, the CGP was mandated to develop governance documents that would outline all policies and procedures relevant to the HeLTI Consortium. In particular, they were tasked with drafting and revising governance frameworks for each cohort and an international framework for the entire consortium. This international governance framework would be informed by and expand upon the cohort frameworks.

As none of the trials had been launched at the time the drafting process was initiated, the ICFs and research protocols served as the starting point for the drafting of the cohort-specific governance frameworks. We, therefore, had flexibility in the creation of templates that would include and account for the various elements required. This would ensure that the governance frameworks could all be harmonized and aligned to facilitate streamlined and interoperable access.

**FIG. 1.**
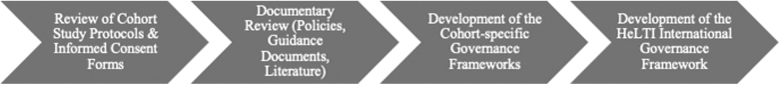
Steps in the development of the HeLTI governance frameworks. HeLTI, Healthy Life Trajectories Initiative.

To inform the drafting process, frameworks and related policies from other longitudinal cohort studies and biobanks were consulted,^16,17^ as were guidance documents on biobank and database governance from organizations, such as the Organisation for Economic Co-operation and Development (OECD) and the Public Population Project in Genomics, and peer-reviewed publications on database and biobank governance.^18–21^ Figure 1 summarizes the steps taken in the development of the governance frameworks.

Importantly, the cohort-specific and international governance frameworks needed to be harmonized to be coordinated and internally consistent. Furthermore, they needed to be sufficiently flexible to allow for potential future amendments. Indeed, throughout the duration of the cohort trials, new scenarios or challenges may arise, such as changes in local laws that may affect data and biosample sharing. The governance frameworks must, therefore, be reviewed periodically to ensure that all clauses are up to date and conform to all relevant normative requirements.

Accordingly, the governance frameworks outline amendment mechanisms, as well as approval processes by relevant bodies. At the international level, this includes approval by representatives from all cohorts. The fundamental elements of the cohort-specific and international governance frameworks are outlined in the following section.

## Results: Fundamental Elements of the HeLTI International and Cohort-Specific Governance Frameworks

### Biobank and database management

Biobanks and databases must have clearly defined and documented procedures for the collection, storage, transfer, use, and destruction of data and biosamples.^22^ Although the protection of participants' rights and interests is primary,^22^ it is essential that the HeLTI cohort-specific databases and biobanks foster future NCD-related research. Accordingly, we aimed to devise biobank and database management procedures that would strike an appropriate balance between these two objectives.^23,24^ For instance, all HeLTI data and biosamples will be coded, which aims to protect research participants by removing direct identifiers, while ensuring optimal scientific utility, which may be lost through complete anonymization.

A key feature of the HeLTI Consortium is that researchers may be granted access to data sets and biosample sets from multiple cohorts. To facilitate sharing and joint analysis, all HeLTI data and biosamples must be interoperable. In other words, they must be able to be shared or exchanged in a facilitated manner. Interoperable data and biosamples are stored and processed under similar analytical conditions that allow researchers “to easily combine them with related items from other biobanks.”^25^ Interoperability is fundamental to international research collaborations, and allows researchers to pool information from research participants of various lifestyle backgrounds and genetic make-ups, resulting in greater statistical power.^25^

However, ensuring standardization between different countries presented certain challenges. In devising uniform and harmonized processes across the cohorts, we needed to be cognizant of the cohorts' different legal and ethical norms, as well as potential scientific, technological, or cultural constraints.^26^ To address these issues, we used internationally accepted database and biobanking standards for the development of processes concerning the collection, processing, handling, and storage of data and biosamples within the HeLTI Consortium, such as OECD's *Guidelines for Human Biobanks and Genetic Research Databases*^22^ and the World Medical Association's *Declaration of Taipei on Ethical Considerations regarding Health Databases and Biobanks*.^27^

All HeLTI data and biosamples will, therefore, be collected in accordance with participants' informed consent, will be coded to remove direct identifiers, and will be stored securely with administrative, physical, and technical safeguards in place to protect participants' privacy and confidentiality. In addition to protecting participants' interests, standardization enhances interoperability, enabling efficient collaboration and interaction between and beyond cohorts.^28^

Research initiatives involving cohorts in multiple countries should consider standardizing processes for database and biobank management by adopting international database and biobanking standards. This will not only help ensure interoperability, which will facilitate the ability to share data and biosamples, but will also help circumvent many of the challenges that are raised when dealing with multiple ethicolegal norms.

### Access policy

The access policy is, arguably, the most important component of the governance framework, as it defines the procedures for data and biosample sharing.^29,30^ An overarching objective of the HeLTI Consortium is to create an infrastructure that allows for responsible data and biosample sharing, as well as harmonized analysis. Recognized as both a scientific and ethical imperative,^31^ data and biosample sharing serves to advance regulatory and policy action, produce new research hypotheses, and foster scientific advancements and benefits.^32^

Nonetheless, data and biosample sharing must be carried out in a responsible manner to maintain public trust in scientific research and to protect the interests of research participants, who benevolently contributed their data and biosamples to research.^31^ Therefore, a balance between protecting participants' interests and promoting public benefits through research must be achieved.^33^ Accordingly, we needed to devise an access policy that would be applicable across all cohorts and coherent with applicable cohort ethicolegal norms. Moreover, the access policy needed to enable informed and efficient collaboration; encourage fair, timely, and transparent access; and ensure that HeLTI data and biosamples would be used in scientifically and ethically validated manners.

From the beginning, given our prior experience in drafting a governance framework for a pan-Canadian population cohort study, we chose to implement a federated data and biosample access system at the international level, rather than separate systems for each cohort.^34^ This federated approach is consistent with other research initiatives involving cohorts from multiple jurisdictions.^35^ In this manner, international researchers who seek access to data and/or biosamples from more than one cohort can apply for access through a centralized process.

Data and biosamples are available for access by both academic and private or commercial researchers. Access procedures are the same for all researchers, whether they are members of the HeLTI Consortium or external researchers, and whether or not they seek access to data and/or biosamples from a sole cohort or from multiple cohorts.

This approach avoids the need for separate access applications to multiple cohorts, which can be a long and administratively burdensome process. In cases where researchers seek access to data and/or biosamples from multiple cohorts, navigating multiple access policies would unduly impede the sharing process. Indeed, it has been noted that “a single centralized application system at a supranational level could greatly improve the efficiency of access to multiple biobanks.”^30^ Creating a “one-stop-shop” procedure, therefore, significantly facilitates and speeds up access processes.

Accordingly, a centralized access portal will be implemented through the HeLTI Office, the body that manages the administrative elements of data and biosample access. Researchers will use this portal to submit all access requests. Where researchers seek access to data and/or biosamples from a single cohort, their access request will be submitted to the appropriate cohort-specific access committee for review. Access committees are the bodies responsible for the review and approval (or refusal) of access requests.^36^

**FIG. 2.**
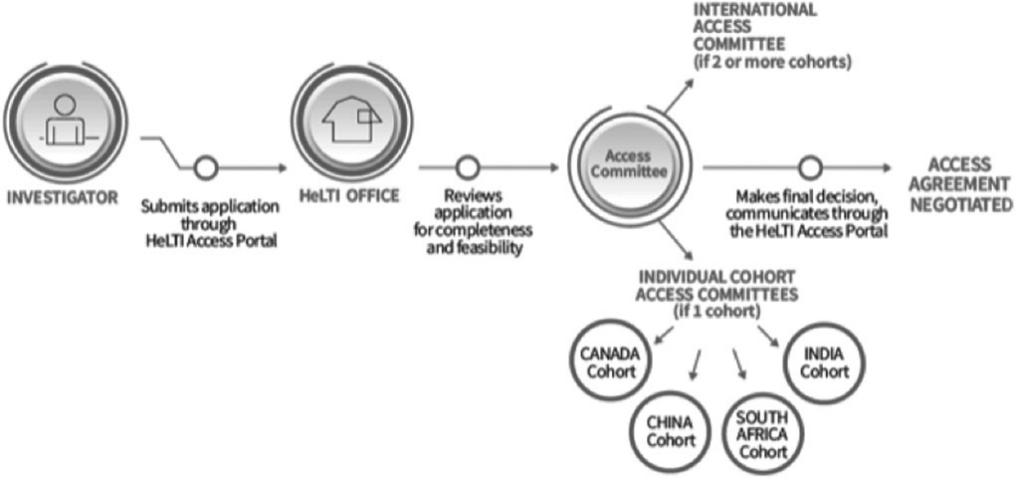
HeLTI Consortium's access process for data and/or biosamples.

In cases wherein access to data and/or biosamples from multiple cohorts is sought, the request will be reviewed by the HeLTI International Access Committee. This streamlines the process by eliminating the need for review by multiple bodies that may reach contradictory decisions. Figure 2 shows the access process and all involved parties.

Although this federated approach serves to facilitate data and biosample sharing across the HeLTI Consortium, it did present certain challenges. As the four cohorts are located in different countries, each jurisdiction has differing laws, regulations, policies, and ethical standards, most notably regarding the export of biosamples outside their respective territorial jurisdictions. China and India, in particular, have clearly defined regulatory mechanisms in place surrounding biosample export that are designed to protect patients, researchers, as well as national interest. The international governance framework, therefore, had to comply with, and be interpreted in accordance with, a complex network of norms.

We needed to account for these normative discrepancies, while still ensuring consistency across cohorts. This was achieved by following the most restrictive cohort-specific legal frameworks—China and India. Since all cohort-specific norms must be respected, and in the spirit of consistency, biosamples will not be allowed to be exported from the cohorts' respective territories, unless authorized by local law in the countries involved. Accordingly, only researchers present in those particular countries will be provided access to biosamples.

Although a federated access approach facilitates data and biosample sharing across countries, local requirements may present certain restrictions that cannot be circumvented. These must be considered and addressed in the governance model. As transparency is an important element in any governance framework, the access policy must reflect and clearly outline these limitations. Transparency is an essential feature of responsible data and biosample sharing and “needs to exist in all workflow” of such sharing activities.^27^

International research initiatives should consider adopting federated access approaches to streamline data and biosample sharing processes. Where local norms potentially restrict the sharing of data and/or biosamples, governance models should follow the most restrictive local normative framework to avoid inconsistent patchwork-style access policies.

### Publication policy

In keeping with the human right to benefit from scientific progress,^37^ researchers who access HeLTI data and/or biosamples are encouraged to share their research results to benefit both the scientific community and society at large.^7^

A publication policy serves to acknowledge the contribution of the database and biobank, increasing their visibility within the scientific community.^7^ Indeed, there exists a consensus in the literature and in data sharing guidelines that recognition systems be implemented to ensure that all parties who contributed to the generation of research results be duly acknowledged.^27^ It was, therefore, important that we include provisions in the cohort-specific and international governance frameworks requiring researchers to acknowledge both the HeLTI Consortium and the cohorts from which data and/or biosamples were accessed.

Prospective cohort studies such as the HeLTI Consortium take many years to establish, collect, and curate their data and biosamples, and involve much investment on the part of the trial investigators and their research teams. The dissemination of research results and knowledge obtained from the use of HeLTI data and/or biosamples must, therefore, acknowledge and respect these contributions.^38^

We considered various approaches of credit attribution to original investigators in the context of population biobanks and databases, including acknowledgment and coauthorship.^39^ Ultimately, we selected the acknowledgment approach identified by Kleiderman et al. as the most common approach in the database and biobanking context.^39^ Under this approach, authors must acknowledge the database(s) and biobank(s) from which they obtained the data and/or biosamples used to generate the research findings presented in their publication.^39^ Furthermore, researchers responsible for the development of specific data or biospecimen collection tools must be invited to collaborate in the proposed project.

This approach was endorsed by the HeLTI Consortium. It requires that researchers acknowledge the consortium and all applicable HeLTI cohorts in publications arising from the use of their data and/or biosamples, as well as other knowledge transfer venues, such as oral or poster presentations. To facilitate this acknowledgment, the cohort-specific and international publication policies include a template paragraph that researchers can modify according to their needs and incorporate into all relevant knowledge translation initiatives.

We decided not to attribute automatic authorship to HeLTI investigators, as it is contrary to the principles of fairness and accountability.^39^ Indeed, authors must respect all of the International Committee of Medical Journals Editors authorship guidelines.^40^ Therefore, authors must (1) have made substantial contributions to the conception or design of the work, (2) have participated in its drafting or revision, (3) had final approval of the version of the work submitted for publication, and (4) agree to be accountable for all aspects of the work.^40^

In developing their own publication policies, international research consortia should consider adopting the acknowledgment approach. It is the most common approach used by population databases and biobanks, aligns with the principles of fairness and accountability in publication ethics, and ensures recognition of the researchers who built these resources and, most importantly, the participants who contributed to them. HeLTI's Publication Policy, therefore, serves as a model from which similar initiatives may draw upon.

### Intellectual property policy

Intellectual property policies are an important component of governance frameworks. They seek to enjoin researchers from appropriating data and biosamples to which they have been granted access, while still promoting collaboration and sharing to advance scientific knowledge.^7^ More specifically, they ensure that data and biosamples are not “exploited improperly or used in a way that inappropriately constrains use” by other researchers.^41^ Such exploitation would be inconsistent with HeLTI's international and collaborative mandate in facilitating data and biosample sharing and with the benefit sharing principle of bioethics.

Similar to our development of the access policy, we adopted the approach used by the Canadian Partnership for Tomorrow's Health Project (CanPath), whereby researchers or their host institutions may not make intellectual property claims on HeLTI data and biosamples.^42^ Allowing researchers to do so would hinder optimal prospective usage of these resources. They may, however, choose to obtain intellectual property rights on subsequent innovations and downstream discoveries. Indeed, intellectual property rights serve to encourage innovation and knowledge dissemination, with “a view to fostering scientific, technical and social progress for the betterment of society.”^43^

One of HeLTI's core principles is that researchers must comply with cohorts' intellectual property legislation. As the HeLTI cohorts are located in four different countries, the intellectual property generated within the context of the HeLTI Consortium will be governed by a multitude of national or local laws and regulations. Although this should not pose any problems in cases wherein access to data and/or biosamples is sought from an individual cohort, it may present certain issues in cases where access to multiple cohorts is requested, as multiple norms will be engaged.

As it was not possible to harmonize these norms, a fragmented approach was taken, whereby researchers will have to comply with cohort-specific intellectual property requirements. As was the case with biosample exportation, compliance with intellectual property norms across cohorts presented a legal limitation to the harmonization of the governance frameworks.

Similar global initiatives should be cognizant of these limitations when developing their intellectual property policies. HeLTI's general approach of precluding intellectual property claims on data and biosamples ensures that these resources are not inappropriately utilized or appropriated. At the same time, allowing such claims on downstream discoveries developed through the use of these resources fosters innovation. This approach, however, may be limited by local norms.

## Discussion: Lessons Learned and Future Recommendations

This article describes our development of the international and cohort-specific governance frameworks of the HeLTI Consortium. Specifically, it provides an overview of the steps that were taken in the development of the key governance elements, with particular emphasis on the key ethicolegal issues relevant to the governance of the HeLTI Consortium: database and biobank management, sharing of data and biosamples, and considerations related to publications and intellectual property. Some components of the governance framework are the same across sites, whereas others depend on cohort-specific considerations. Table 1 illustrates these components.

**Table 1.** Similarities and Differences Across Cohorts in the Healthy Life Trajectories Initiative Governance Frameworks

	Same for all cohorts	Different across cohorts
Database and biobank management	- *Similar policies and standards for data and biosample collection, storage, and safeguards*	
Access policy	- *Centralized access process for data and/or biosamples for multiple cohorts*	- *Individual access process for data and/or biosamples from one cohort* - *Regulatory limitations on biosample exportation in certain cohorts*
Publication policy	- *Acknowledgment of HeLTI Consortium and cohorts from which data and/or biosamples were accessed*	
Intellectual property policy	- *General approach: preclusion of intellectual property claims on data and biosamples. Intellectual property claims on downstream discoveries and innovations permitted*	- *Local intellectual property laws may supersede general approach*

HeLTI, Healthy Life Trajectories Initiative.

Unlike other NCD-related research initiatives, which have mainly focused on collaborations between researchers within one country, HeLTI is an international research collaboration, linking researchers across four countries with the common goal of informing policy and decision making for the treatment and prevention of NCDs. In developing the governance frameworks for this initiative, we learned a number of lessons from which future international research collaborations may draw upon in developing their governance models.

The first and perhaps most important lesson was that of harmonization of standards, policies, and procedures across cohorts. Ensuring harmonization is key to the long-term operation and sustainability of collaborative research projects. This is especially pertinent when it comes to database and biobank management and access to data and biosamples. Harmonizing management policies, including those related to the collection and storage of data and biosamples, ensures interoperability of resources between cohorts. Moreover, adopting a centralized federated access process significantly streamlines matters, allowing data and samples to be more easily shared for research.

A second related lesson was that of accounting for local legal and normative standards. Although we were largely successful in ensuring harmonization across cohorts, we encountered certain challenges regarding compliance with country-specific norms, specifically regarding biosample exportation and intellectual property considerations. The potential for local norms to supersede some of the provisions of the governance frameworks needed to be clearly reflected. These norms, which can change over time, should be considered in any future international governance development.

Accordingly, our final key lesson was that of constructing the governance frameworks as living long-term documents, which may change over time in light of changing norms or new practical challenges that may arise during the course of the research. Governance frameworks should, therefore, not be construed as static instruments, but rather flexible documents that can change over time to reflect and respond to the changing realities of the research.

## Conclusion

As scientific research becomes increasingly global and collaborative, developing governance models will likely present numerous challenges. The HeLTI governance frameworks may serve as models for future initiatives that may encounter such challenges as they move from national to international dimensions. The key lessons learned in the development of the governance frameworks may also serve to foster greater collaboration among researchers from different studies around the world and inform cross-national policy consideration in research.
